# A nasolabial cyst is It a rare cyst or not? case series of bilateral and unilateral patterns

**DOI:** 10.3389/froh.2025.1721505

**Published:** 2025-12-17

**Authors:** A. B. Alolayan, S. Alzahrani, D. Alsuwied, A. Jaafar, E. Essa

**Affiliations:** 1 Department of Oral and Maxillofacial Diagnostic Sciences, College of Dentistry, Taibah University, Madinah, Saudi Arabia; 2 Department of Oral and Maxillofacial Surgery, College of Dentistry, King Abdulaziz University, Jeddah, Saudi Arabia; 3 Department of Dentistry King Faisal Specialist Hospital and Research Center-Jeddah Al Rawdah Road, Jeddah, Saudi Arabia; 4 College of Medicine, Alfaisal University, Riyadh, Saudi Arabia; 5 Faculty of Dentistry, Tanta University, Tanta, Egypt

**Keywords:** bilateral lesions, case series, nasolabial cyst, non-odontogenic cysts, unilateral

## Abstract

**Background:**

Nasolabial cyst (NLC) is an extraosseous anterior maxillary cystic lesion located lateral to the midline. It is of epithelial, non-odontogenic origin and is considered a rare pathology. It has a controversial etiopathogenesis, which is still under debate. Commonly presenting as a slowly growing unilateral asymptomatic swelling of the alar region, it may obliterate both the nasolabial fold and maxillary labial sulcus, causing alar nasal elevation. Bilateral cases are rare. Facial asymmetry is the main complaint, prompting patients to seek treatment. Various imaging techniques, in addition to histopathological examination, can confirm the presence of the cyst. Surgical excision is the treatment of choice.

**Case presentation:**

This case series outlines bilateral and unilateral NLC cases in which the patients sought medical care for painless facial swelling. Comprehensive diagnostic methods confirmed the diagnosis of an NLC. Surgical enucleation was performed, resulting in complete resolution of the lesions, with no signs of recurrence.

**Conclusions:**

The represented cases illustrate a holistic description of the NLC features, diagnosis, and treatment, emphasizing the importance of understanding this cyst. Briefly reviewing the literature, they highlight the significance of reinvestigating the true prevalence of the NLC

## 1 Introduction

Nasolabial cyst (NLC) is an uncommon, extraosseous, non-odontogenic maxillary cyst. It comprises 0.7% of all maxillofacial cysts and approximately 2.5% of the maxillofacial non-odontogenic cysts (1). This benign soft tissue cyst is located between the nasal vestibule and the upper lip in a unilateral pattern 90% or, less commonly, in a bilateral pattern 10% (1). The first case was reported by the Austro-Hungarian anatomist Emil Zuckerkandl in 1882 (1, 2). Various nomenclatures have been used to describe NLC, among which are Klestadt's cyst, in honor of Klestadt, who studied it extensively in 1953 (3), and nasoalveolar cyst, as it can involve erosion of the maxillary alveolar bone (4). In 1951, Rao introduced the accurate terminology: NLC, describing the purely soft tissue cyst occupying the nasolabial region (1, 2). The typical demographics of NLC are Black or East Asian females in the fourth to fifth decades of life (1, 2). This localized slowly growing cyst ranges in size from 1 cm to 5 cm and can grow in three directions: toward the nasolabial fold, toward the nasal vestibule and vestibule of the mouth (5). NLC may be asymptomatic, but when it is not, the three main symptoms are nasal obstruction, localized swelling and/or local pain (6).

Diverse unilateral NLC cases have been published in the literature, and only a limited number have reported bilateral cases. In this paper, we present a comprehensive description of two NLC cases as a case series: a uniquely large bilateral case with an overview of the cyst features, diagnosis and treatment approaches, to further familiarize professionals with this rare entity; and a unilateral cyst, with insights into the significance of reinvestigating the true prevalence of unilateral NLC.

## 2 Case description

### 2.1 Case 1: bilateral NLC

A 40-year-old female patient, with no known systemic comorbidities, presented to the Oral and Maxillofacial Surgery Department at the College of Dentistry in Tanta University, Egypt. Her chief complaint was painless, bilateral swelling below the alae of the nose, which caused facial asymmetry. The swelling began 4 years earlier and had increased gradually. In addition, the patient had partial nasal obstruction on the right side. She had not undergone any previous consultation or intervention.

**Figure 1:**
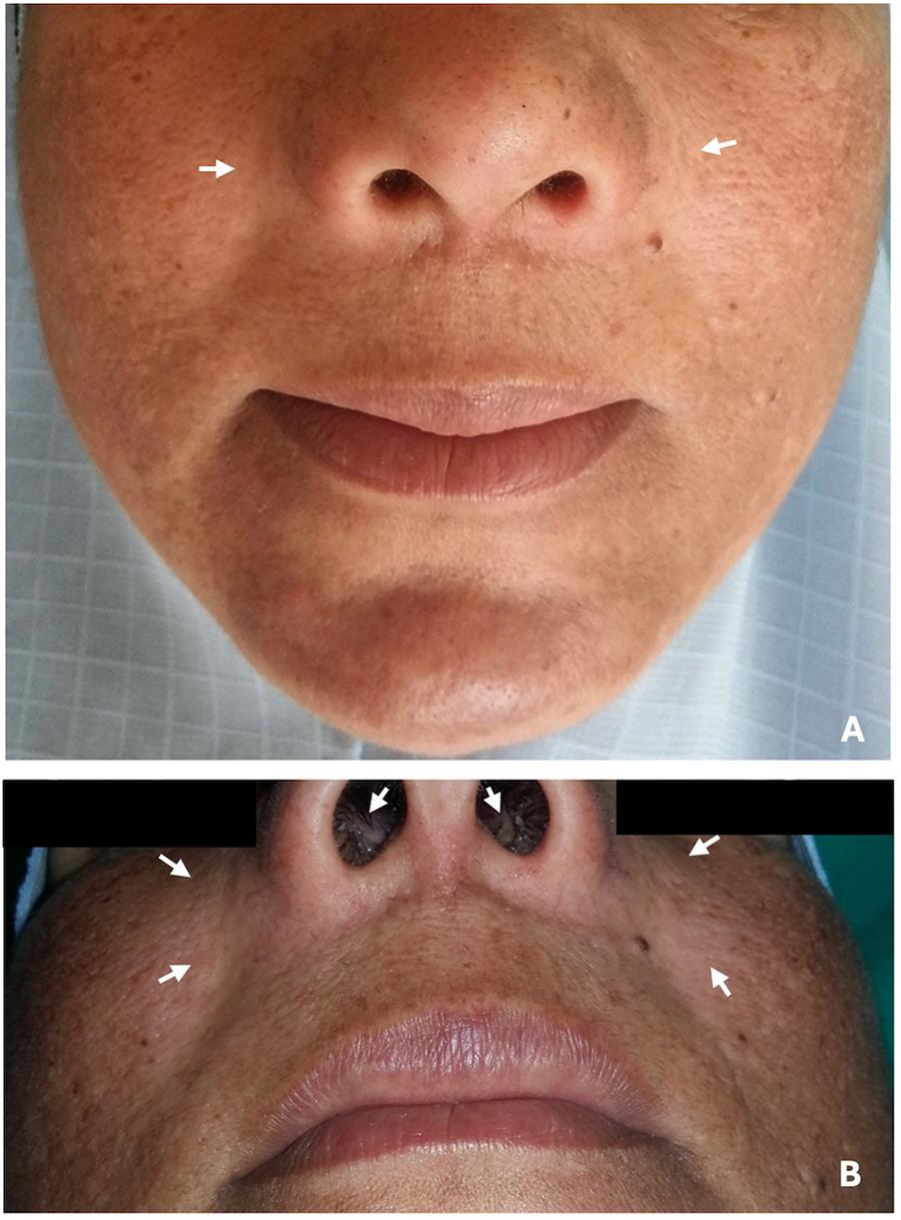
Frontal view showing **(A)** bilateral extraoral swelling below alar nasal region and obliteration of nasolabial fold, **(B)** partial obstruction of both nostrils and bilateral elevation of ala of the nose (arrows).

An extraoral examination revealed a painless, diffuse, bilateral swelling in the nasal alar region, with bilateral obliteration of the nasolabial fold, a bilaterally elevated alar base, and obstruction of both nostrils, as shown in Figures 1A,B. During bimanual palpation, the swelling appeared soft in consistency, fluctuant, and nontender.

A soft, bilateral, fluctuant swelling, covered by normal mucosa, was observed on intraoral examination. It extended from the superior vestibular fornix toward the nose, causing obliteration of the labial vestibule at the region of tooth 13, contralateral to tooth 23.

**Figure 2:**
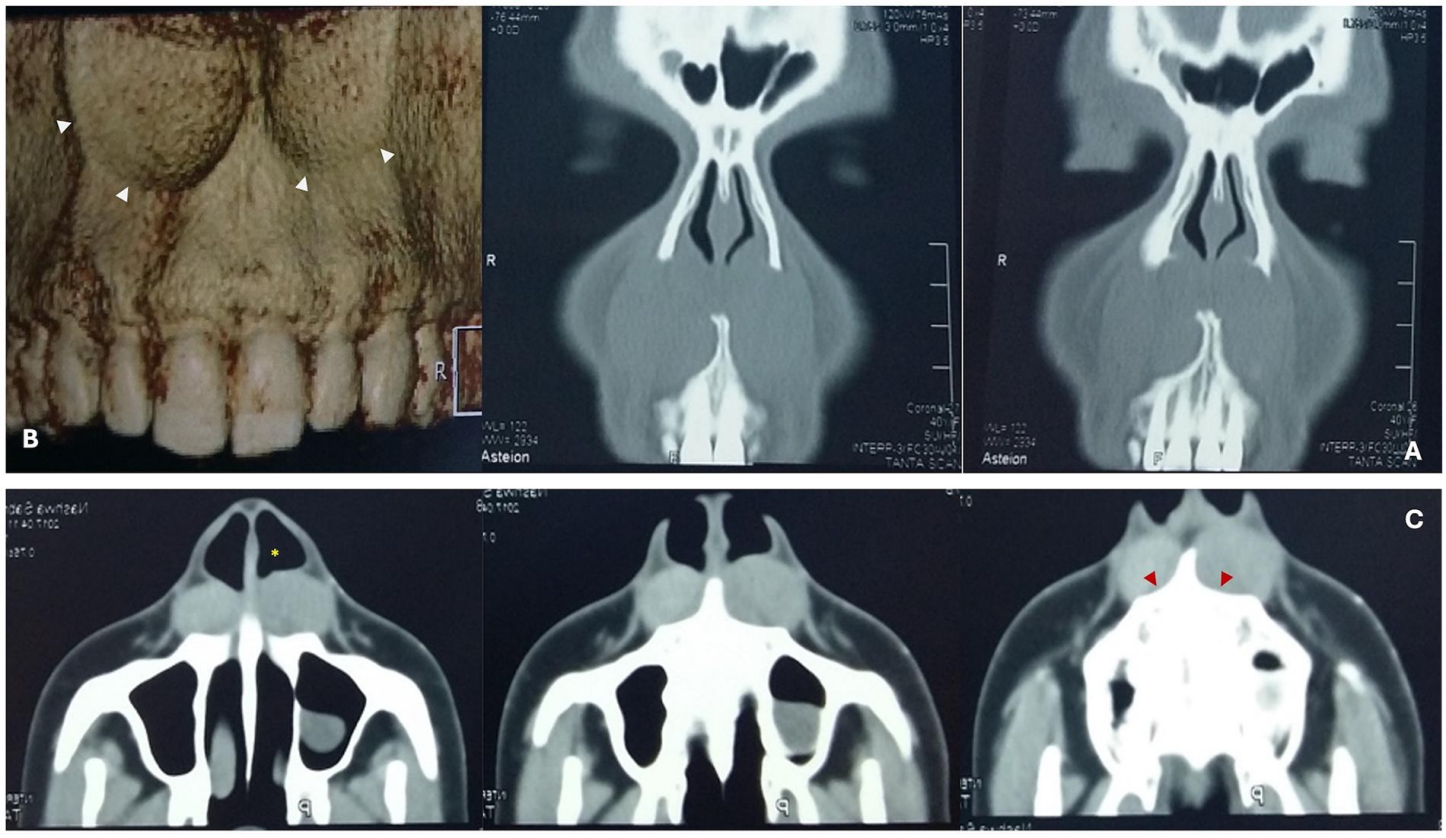
A paranasal sinus CT photo-radiographs showing **(A)** coronal view with partial obstruction of both nostrils **(B)** axial soft tissue view has bilateral well defined extraosseous hypodense, homogeneous oval lesion (red arrowheads: smooth scalloping of maxillary alveolar bone, yellow asterisk: narrowing of the pyriform aperture) **(C)** 3D CT scan demonstrates bilateral pressure bone resorption causing scalloping pattern of the anterior maxillary alveolar bone (arrowheads).

A panoramic radiograph showed no abnormality in the anterior maxillary region, and the related teeth were vital and intact. Axial and coronal CT images revealed a well-circumscribed, hypodense texture of two oval lesions extending from the lateral wall of the nasal cavity. The right lesion measured approximately 4.1 × 3.28 × 4.43 cm, while the left lesion measured around 3.73 × 2.7 × 4.45 cm. The three-dimensional (3D) CT scan showed no anterior maxillary bone resorption. However, it did reveal a bilateral scalloping pattern of the alveolar bone due to the mass effect on the maxilla, as shown in Figures 2A‒C.

From the above clinical and radiographic findings, a diagnosis of bilateral NLC was recorded. The operative decision was to enucleate the lesions bilaterally under general anesthesia.

**Figure 3:**
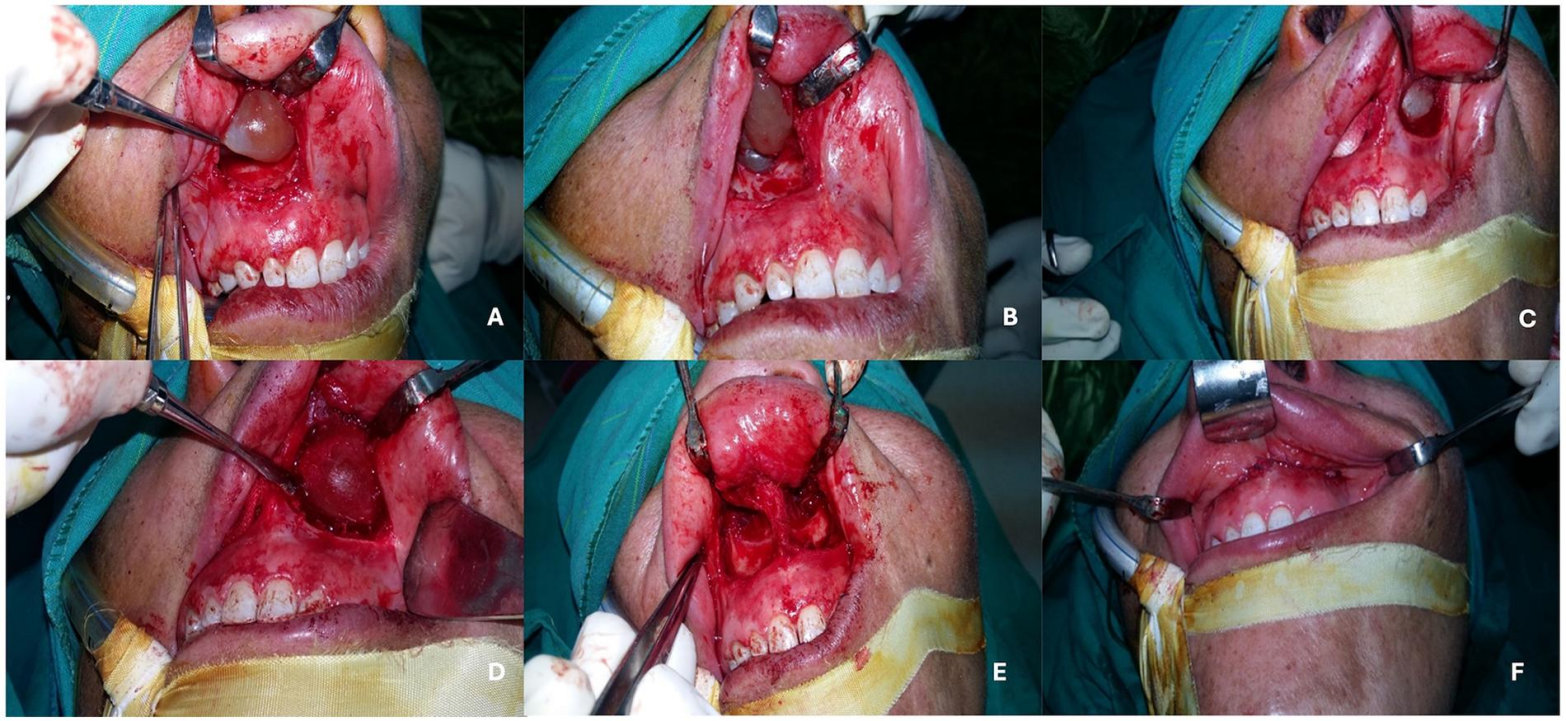
Intraoperative photograph showing **(A)** blunt dissection of right nasolabial cyst, **(B)** complete exposure and preservation of nasal mucosa intact, **(C)** left sublabial approach, **(D)** adequate exposure of the left nasolabial cyst, **(E)** bilateral enucleation and hemostasis and **(F)** closure using continuous with lock vicryl suturing technique.

First, an intraoral sublabial incision of 2 cm was performed on the right side in the upper vestibular sulcus, 1 cm above the superior margin of the attached gingiva. A blunt submucosal dissection was performed, ensuring that tearing or rupturing of the cyst lining was avoided. Following complete exposure of the cyst lining, and because of the intimate adherence to the nasal mucosa, a careful dissection of the cystic wall from the thin and friable nasal mucosa was performed. Meticulous enucleation of the right NLC was also achieved, leaving behind intact overlying nasal mucosa. After hemostasis of the wound had been completed, the same procedure was performed on the left side. The surgical wound was closed using a continuous interlocking technique with Vicryl absorbable sutures, as shown in Figures 3A–F. The surgical specimen was sent to the college's oral pathology laboratory for histopathological examination.

**Figure 4:**
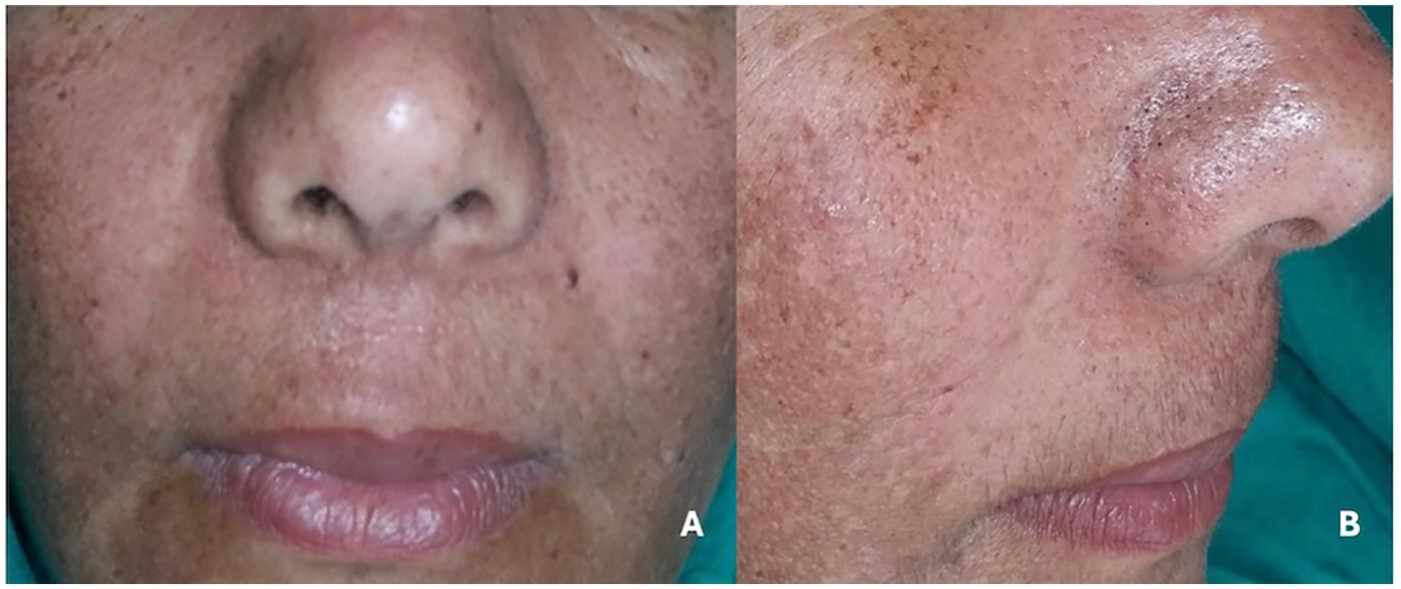
**(A,B)** postoperative photograph showing uneventful soft tissue healing and improvement of extraoral frontal and profile views.

The patient visited for consultation, and radiographic imaging was taken at the same visit. Surgery was performed ten days after the first visit, and the patient returned for a follow-up a week later. Following the operation, she showed excellent, uneventful soft-tissue healing and marked improvement of extraoral asymmetry, as shown in Figures 4A,B. The patient was satisfied with regaining facial symmetry and the resolution of her partial nasal obstruction.

### 2.2 Case 2: unilateral NLC

A 56-year-old female patient, with no known systemic comorbidities, was referred to the oral and maxillofacial surgery department for surgical management of intraoral soft-tissue swelling in the edentulous left anterior maxillary region and for the removal of remaining roots to allow construction of a complete denture. The patient's chief complaint was an inability to eat properly, in addition to mild facial asymmetry due to painless swelling below the left nasal ala region, which had been noticed two years earlier. No previous consultation or intervention took place.

**Figure 5:**
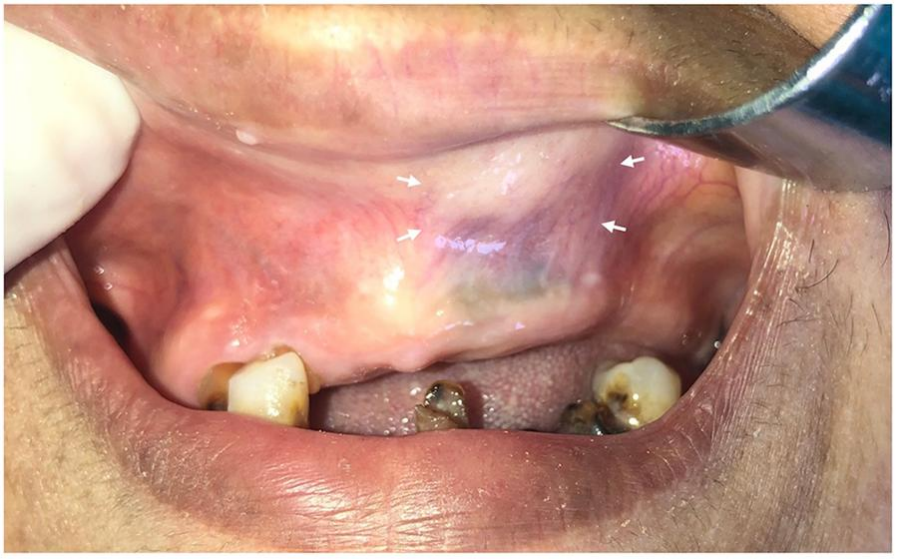
Preoperative intraoral photograph showing left anterior extraosseous swelling with obliteration of the labial fold (arrows).

Extraoral examination revealed mild facial asymmetry and partial obstruction of the left anterior nasal cavity, while intraoral examination showed a painless, nontender, soft, sublabial, and fluctuant extraosseous swelling measuring 2 cm, with partial obliteration of the left labial fold, as shown in Figure 5. Aspiration was performed, and a viscous, yellow fluid was obtained.

**Figure 6:**
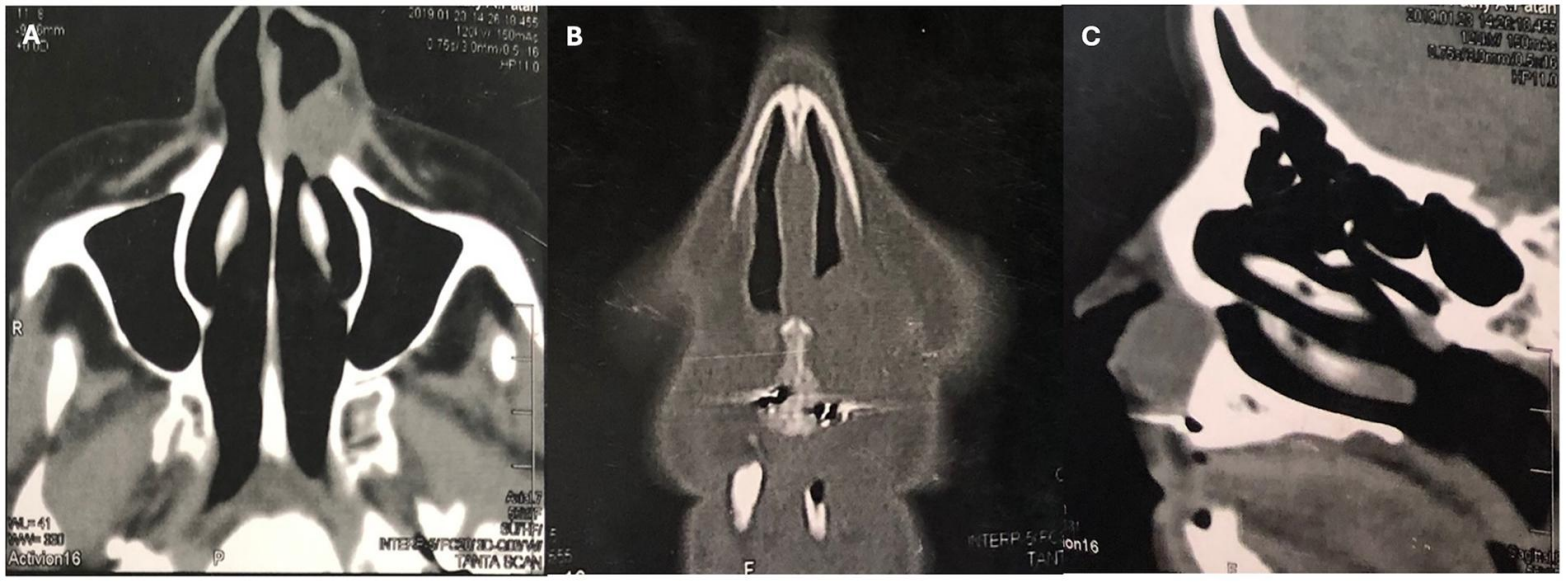
A paranasal sinus soft tissue CT photo-radiographs showing **(A)** axial view with unilateral left well defined extraosseous round lesion with close proximity to nasal cavity, **(B)** coronal view with partial obstruction of left nostrils and **(C)** sagittal view illustrate round lesion encroached up on anterior maxillary alveolar bone without resorption.

Axial, coronal, and sagittal CT soft-tissue scans showed a cystic lesion in the left lateral nasal region, measuring approximately 2.25 × 2.81 × 2.73 cm. Based on the clinical and radiographic findings, a provisional diagnosis of NLC was made, as shown in Figures 6A–C.

**Figure 7:**
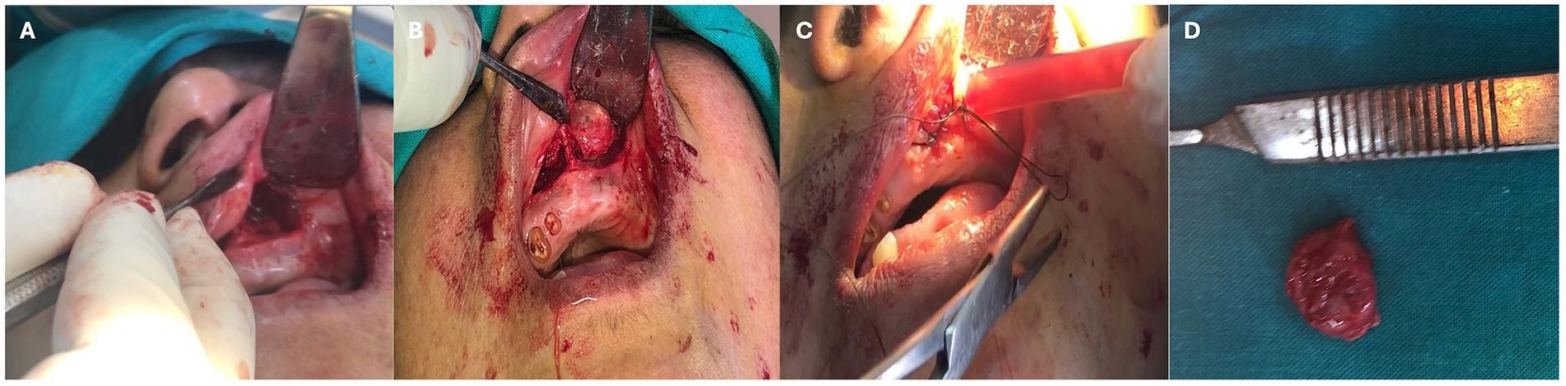
Intraoperative photograph showing **(A)** complete exposure of left nasolabial cyst, **(B)** enucleation of the entire cystic wall and hemostasis, **(C)** closure using continuous with lock suturing technique, **(D)** the enucleated specimen.

The patient visited for consultation, and radiographic imaging was taken at the same visit. Surgical management was performed one week later, and the patient returned for follow-up a week later. The cyst was enucleated via an intraoral, full-thickness mucosal, sublabial approach below the pyriform apertures, using an infraorbital nerve block with local anesthetic, and a submucosal infiltration of 2% mepivacaine hydrochloride with 1:20,000 levonordefrin was used for hemostasis. The cyst lining was detached from the oral tissues bluntly, along with enucleation of the cyst and complete removal of its entire wall. The wound was closed using a continuous interlocking technique, as shown in Figures 7A–D. The surgical specimen was sent to the college's oral pathology laboratory for histopathological examination.

### 2.3 Histopathological findings

**Figure 8:**
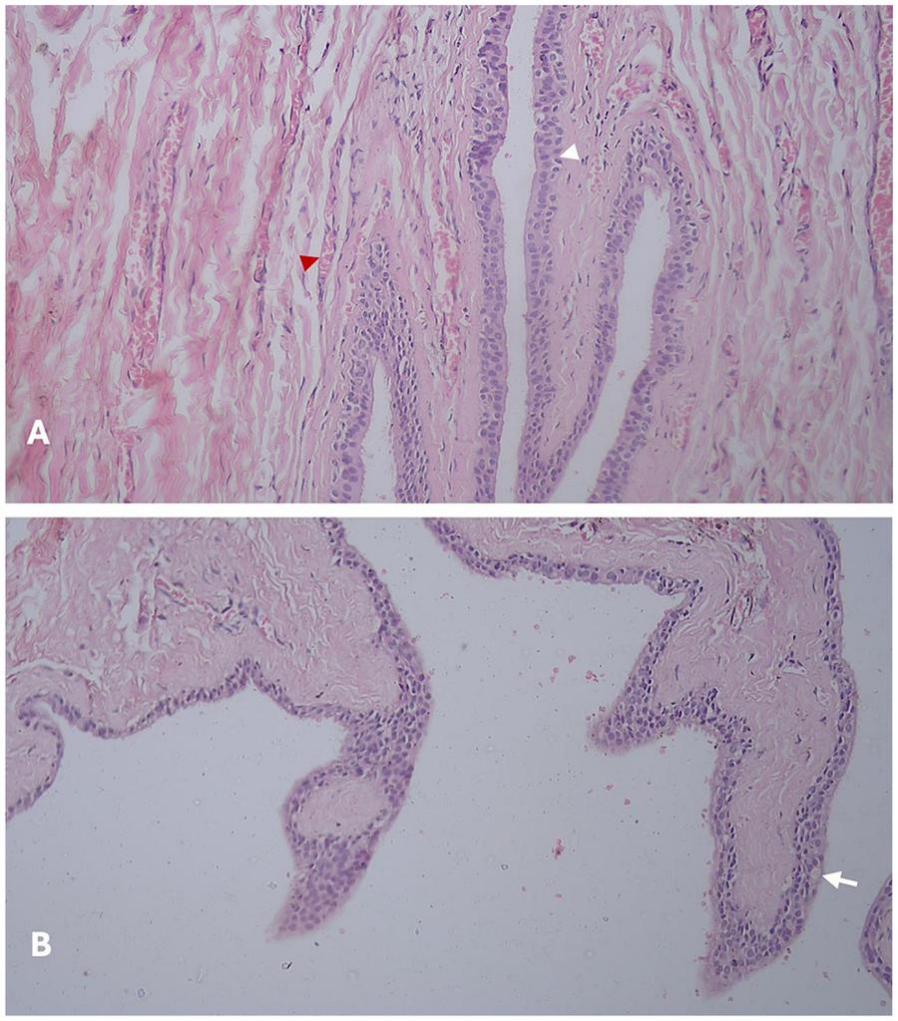
H&E-stained sections of nasolabial cyst, 40× magnification **(A)** white arrowhead: pseudostratified columnar epithelium lining thin cystic wall, red arrowhead: fibrovascular connective tissue stroma, **(B)** white arrow: intraepithelial goblet cells.

Macroscopic examination of the excised specimens revealed soft- to firm-textured masses with a smooth surface and yellow-tinged seromucous contents. The gross description of the first excised biopsy from the bilateral case revealed a consistency of soft- to firm-textured mass, light brown in color, while the undersurface was lobulated with a white cut surface of irregular thickness. The microscopic hematoxylin and eosin (H&E)-stained tissue sections showed a cystic cavity lined by ciliated, pseudostratified columnar epithelium containing intraepithelial goblet cells. The underlying connective tissue wall was dense and fibrous, and there was mild infiltration by chronic inflammatory cells, as shown in Figure 8A. The gross description of the second excised biopsy from the unilateral case revealed a consistency of firm-textured mass, yellowish-brown in color, while the microscopic H&E-stained tissue sections showed a cystic cavity lined by respiratory epithelium, consisting of pseudostratified and stratified columnar epithelium with goblet cells. The underlying connective tissue was densely infiltrated with chronic inflammatory cells, as shown in Figure 8B.

## 3 Discussion

The diagnosis of the rare NLC is based on clinical examination, radiographic findings, and key symptoms, but a definitive diagnosis is confirmed through histopathological results (3, 5, 7). Clinically, the location of the NLC in the submucosa of the anterior nasal floor is highly diagnostic, and it was considered pathognomonic by Bull et al. in 1967 (2, 7). Another distinctive clinical finding is the fluctuant cystic consistency upon bimanual palpation, using two fingers—one on the nasal floor and one in the labial sulcus (2, 4–6)—which was confirmed in both of our cases. Although plain radiographs are of limited significance in NLC cases, being used only to exclude other odontogenic or non-odontogenic lesions (1, 4), ultrasonography, CT, and MRI are highly diagnostic (1, 5, 7, 8). Ultrasonography is an office-based, cost-effective, short-duration examination used in the diagnosis of NLC, which can accurately detect the cyst wall and contents (7, 8). It is the main diagnostic radiography for patients with metal implants because metal artefacts can interfere with the diagnosis of lesions in CT and MRI (8). However, as this exam is operator-dependent, the accuracy of the results depends on the level of experience (5). CT scanning has been reported as the imaging modality of choice, and therefore, it is imperative in NLC preoperative assessment (1, 5, 7). NLC is detected on CT scan as a well-defined, rounded, homogeneous, low-density soft tissue lesion at the alar base and lateral to the piriform aperture. The compressive effect of the cyst can cause bone remodeling, which appears as scalloping of the underlying maxillary bone (1, 5–7). This typical CT appearance was also detected in our reported cases. Compared to CT, MRI has a higher soft tissue contrast resolution, and it is superior in detecting cyst origin and contents, but cost can be a limitation (6–8).

The pathogenesis of NLC has been explained by various theories, with the most widely accepted theory proposed by Bruggemann in 1920, stating that NLC develops from epithelial remnants in the inferior anterior part of the nasolacrimal duct (2, 4, 5). This theory is reinforced by the consistent location of the cyst at the anterior nasal floor and by the fact that the most frequently observed histopathological pattern of the NLC lining is compatible with the ductal or glandular epithelium lining of the nasolacrimal duct (4, 5, 9). Classic histopathological findings of NLC show fibrous capsule, signs of chronic inflammation, and a multilayer cystic lining of ciliated pseudostratified columnar epithelium, or in some areas stratified squamous epithelium, with goblet cells (4–6, 9). These findings were observed in both of our cases, confirming the preliminary diagnosis of NLC and pointing towards a shared embryonic origin regardless of laterality. Besides this theory, some authors reported the role of triggering factors, such as a history of trauma and infection, in stimulating or hastening the formation of the cyst from dormant epithelial remnants (7, 10). This fact is emphasized by NLC development in patients with a history of cleft repair surgeries or recurrent dacryocystitis (11–13). Similarly, Ozdogan et al. detected a bilateral NLC after rhinoplasty and alar base reduction surgery, supporting the involvement of both surgical and non-surgical trauma in cyst development (14).

The differentials of the NLC include both odontogenic and non-odontogenic lesions. Odontogenic lesions include periapical cysts, granulomas, abscesses, and dentigerous cysts with cortical perforation (9, 15). These are osseous lesions that can be easily distinguished by routine radiographs, showing dental origin, and by pulp vitality testing (3, 9, 15). Mostly, an abnormal vitality test response indicates the presence of dental inflammatory condition. Nevertheless, Martini et al. documented an NLC communicating with tooth apices in which the related teeth responded negatively to pulp test, and the lesion was initially misdiagnosed as a dental abscess (3). Common non-odontogenic differentials are incisive canal cyst, globulomaxillary cyst, dermoid cyst, and epidermoid cyst. Nasopalatine duct cyst is an intraosseous anterior midline lesion, and globulomaxillary cyst is a fissural intraosseous cyst, classically located between the lateral incisor and canine (9). Epidermoid cyst is covered with yellowish overlying mucosa due to its sebaceous content, compared to the normally colored or bluish mucosa covering NLC (3, 9). Dermoid cyst is located at the midline or in the medial canthal region (9). Both are usually detected in childhood (3, 9) and histopathologically are lined by keratinized stratified squamous epithelium like epidermis, with evidence of dermal appendage structures within the dermoid cyst wall (1). In case of an infected NLC, it can resemble facial cellulitis, periodontal abscess, nasal furuncle, and maxillary sinusitis (5). Other less common differentials include nasolacrimal mucoceles seen in infants, and unilocular lymphangioma, which has similar imaging findings to those of NLC (9). Schwannoma is a benign neoplasm which can mimic the features of NLC when located beneath the nasal ala and should be considered in the differential of nasolabial swellings (15). Rai et al. reported a case of ameloblastoma in the nasolabial region, which was initially misdiagnosed radiographically as NLC, and concluded that ameloblastoma and its rare malignant variant must be considered during the diagnosis of nasolabial swellings (7). This wide variety of differentials alerts practitioners to the importance of knowledge about NLC.

Clinically, NLC involves at least one of the key symptoms: nasal obstruction, localized swelling, or localized pain (6). Difficulty in nasal breathing, nasal blockage, and rhinorrhea can be observed depending on the degree of nasal obstruction caused by the cyst, especially when in close proximity to the inferior turbinate, which can be misdiagnosed as inferior nasal turbinate hypertrophy (1, 5). Localized swelling caused by NLC leads to facial deformity, as it obliterates the nasolabial fold and elevates the nasal alae (1). Pain is usually associated with an infected NLC if the cyst ruptures, drainage into the nose or mouth can be observed (1). Considering NLC location between the nasal cavity and teeth, it can easily develop an infection (16). Although not typical, NLC can lead to tooth displacement due to bone erosion and invasion of supporting structures around related teeth (4). If NLC compromises lacrimal drainage, especially in bilateral cases, dacryocystitis and epiphora may develop (1, 17).

Treatment modalities include surgical enucleation, transnasal endoscopic marsupialization, sclerotherapy, incision and drainage, and aspiration (1, 2). Recurrence depends on treatment approach; surgical resection and marsupialization have recorded a good long-term prognosis and rare recurrence (2, 18). Transnasal endoscopic marsupialization showed short operative time, minimal intraoperative bleeding, and no postoperative pain or edema. It is based on the concept of converting the cyst into an air-containing sinus at the anterolateral nasal floor (2, 18). However, if the created window is small, it can lead to shrinkage of the scar around the ostium, with subsequent mucus accumulation or cyst recurrence (2). Surgical resection is both curative and diagnostic; it is performed via a sublabial incision or Neumann incision. Postoperative complications, such as oronasal fistula, swelling, hematoma, numbness in teeth, and wound infection, can develop (2, 18). Herein, the bilateral NLC case showed a technical challenge: the large cyst size and intimate contact between the cyst wall and nasal mucosa required precise dissection to avoid laceration of the nasal mucosa and subsequent oronasal fistula formation.

NLC has two patterns: the more common unilateral pattern and the rarer bilateral pattern. In 2016, Sato et al. reported a summary of published bilateral cases since 1967, showing only 18 cases (10). Additionally, Liu et al.'s study analyzed data from 20 NLC cases from 2021 to 2022, among which only one case showed a bilateral pattern (8). Another retrospective study completed in a tertiary care center analyzing 38 NLC cases treated between 2018 and 2022 reported only three bilateral cases (19). The documented size of the NLC ranges from 1 cm to 5 cm. In this paper, the rare bilateral pattern and the large size of the cyst make this case particularly noteworthy. A brief overview of the literature revealed only one other unilateral large NLC reported by Ordones et al. in 2013 (16). Notably, in our case, there was a size difference between the left and right lesions; the growth rate of bilateral cysts can be asynchronous, leading to a varying degree of cosmetic disruption between the sides. Knowledge about NLC and other nasolabial swelling differentials is imperative to avoid misdiagnosis and unnecessary treatment. Several studies have documented initially misdiagnosed NLC, and some have shown other lesions resembling NLC presentations (7, 15). A case report documented a mistreatment, which led to the loss of a tooth because of misdiagnosing NLC as a periapical abscess (20). A similar misdiagnosis caused a patient to suffer for several years, as symptoms persisted after multiple unnecessary endodontic treatments and repeated scaling (21). Moreover, besides typical documented features, NLC continuously shows variability, e.g., unusual location in the upper lip clinically diagnosed as a lipoma (22), occurrence in an 11-year-old child (23), and incidental finding of NLC, causing nasal bone displacement and repulsion in the turbinate without external soft tissue swelling, during septorhinoplasty to treat nasal deformation and turbinate hypertrophy (24). All aforementioned points further emphasize the importance of documenting NLC cases and knowledge about this entity.

Originally, NLC was presented as a rare entity; however, over the years, various unilateral NLC cases have been published. Several authors suggested that NLC occurrence is greater than what is documented in the literature (2, 5). This uncertainty about the true prevalence may be attributed to various factors. The fact that NLC can be treated by different specialists (2), OMFS (10–12), ENT (2, 5, 6), and plastic surgery (24), could affect the accuracy of reported prevalence. Another factor is that NLC's prevalence differs across various regions of the world, being relatively low in the Western world and higher in Asian and African American (10). Most of the published case reports emerged from Asian countries (India (19), China (8), Japan (10), Turkey (18) and some from African countries (Egypt (25), Morocco (26) and Latin America (Brazil (3); the population size and access to healthcare in these regions should be considered. Also, advances in medical imaging and the level of awareness may have impacted the detection rate of NLC. Compared to 18 cases detected in a study from 1988 to 1999 (27), more recent studies found 20 and 38 NLC cases in one year and over a four-year interval, respectively (8, 19). Likewise, a prospective study performed to explore the effectiveness of laser transnasal marsupialization registered 12 NLC patients in one year (28). Another example, a study reported that 11 patients with nasolabial swellings visited between 2018 and 2020, ten of whom were found to have NLC (25). Therefore, these insights and others shed light on the question about the true prevalence of NLC.

In conclusion, NLC should be considered in the nasolabial swelling differentials. Knowledge about both patterns is significant for timely diagnosis and appropriate treatment to avoid complications, misdiagnosis and unnecessary interventions. Prevalence of unilateral NLC could be reassessed on a larger scale across different regions.

## Data Availability

The raw data supporting the conclusions of this article will be made available by the authors, without undue reservation.

## References

[1] Toribio Y Roehrl M. The nasolabial cyst: a nonodontogenic oral cyst related to nasolacrimal duct epithelium. Arch Pathol Lab Med. (2011) 135(11):1499–503. 10.5858/arpa.2010-0338-RS 22032581

[2] Almutairi A Alaglan A Alenezi M Alanazy S Al-Wutayd O. Nasolabial cyst: case report and review of management options. BMC Surg. (2020) 20(1):10. 10.1186/s12893-020-0677-3 31924189 PMC6954569

[3] Martini EC Coppla FM Campagnoli EB Bortoluzzi MC. Nasolabial cyst associated with odontogenic infection. Case Rep Dent. (2016) 2016:8690593. 10.1155/2016/8690593 26904312 PMC4745964

[4] Parwani R Parwani S Wanjari S. Diagnosis and management of bilateral nasolabial cysts. J Oral Maxillofac Pathol. (2013) 17(3):443–6. 10.4103/0973-029X.125217 24574670 PMC3927353

[5] Swain SK. Nasolabial cyst: a narrative review. Matrix Science Medica. (2023) 7(2):23–7. 10.4103/mtsm.mtsm_13_22

[6] Tiago RS Maia MS Nascimento GM Correa JP Salgado DC. Nasolabial cyst: diagnostic and therapeutical aspects. Braz J Otorhinolaryngol. (2008) 74(1):39–43. 10.1016/s1808-8694(15)30749-7 18392500 PMC9452201

[7] Rai K Carvalho C Sagane B Velankar HK Agrawal S Hawkes G. Questionable nasolabial lump: a case report. J Adv Med Med Res. (2020) 32(10):38–44. 10.9734/jammr/2020/v32i1030517

[8] Liu S Hao D Yu L Ma H Zhao H Sun S Comparative analysis of three common imaging modalities for nasolabial cysts. J Int Med Res. (2023) 51(1):3000605221147201. 10.1177/03000605221147201 36597377 PMC9830098

[9] Patil AR Singh AP Nandikoor S Meganathan P. Bilateral nasolabial cysts—case report and review of literature. Indian J Radiol Imaging. (2016) 26(2):241–4. 10.4103/0971-3026.184424 27413273 PMC4931785

[10] Sato M Morita K Kabasawa Y Harada H. Bilateral nasolabial cysts: a case report. J Med Case Rep. (2016) 10(1):246. 10.1186/s13256-016-1024-2 27604349 PMC5015322

[11] Suenaga H Hosokawa R Saijo H Hoshi K Takato T. Nasolabial cyst in a patient with cleft lip and alveolus: a case report. J Oral Maxillofac Surg Med Pathol. (2015) 27(6):839–42. 10.1016/j.ajoms.2015.04.009

[12] Fyrgiola M Lianou V Katoumas K Nikitakis N. Nasolabial cyst in a patient with cleft lip and recurrent dacryocystitis. J Oral Maxillofac Surg Med Pathol. (2019) 31(5):365–8. 10.1016/j.ajoms.2019.02.008

[13] McClain MW Miller M Varman R. Delayed onset perinasal mucocele cyst in a patient with remote history of orofacial cleft repair. J Craniofac Surg. (2025) 36(3):e283–6. 10.1097/SCS.0000000000010863 39509732

[14] Ozdogan F Ozel HE Esen E Yuce T Baser S Yavuz CS. An unexpected rhinoplasty complication: bilateral nasolabial cyst. Aesth Plast Surg. (2015) 39(6):888–91. 10.1007/s00266-015-0564-y 26392372

[15] Iida S Aikawa T Kishino M Sakai T Nakano Y Okura M Spheric mass beneath the alar base: MR images of nasolabial cyst and schwannoma. AJNR Am J Neuroradiol. (2006) 27(9):1826–9. PMID: . 17032851 PMC7977907

[16] Ordones AB Neri L Oliveira IH Tepedino MS de Rezende Pinna F Voegels RL. Giant nasolabial cyst treated using neumann incision: case report. Int Arch Otorhinolaryngol. (2013) 17(4):421–3. 10.1055/s-0033-1351674 25992051 PMC4399185

[17] Kyrmizakis DE Lachanas VA Benakis AA Velegrakis GA Aslanides IM. Bilateral nasolabial cysts associated with recurrent dacryocystitis. J Laryngol Otol. (2005) 119(5):412–4. 10.1258/0022215053945787 15949112

[18] Cebi IT Karataş A Yüce T Şalvız M Koçak A Selçuk T. Bilateral nasolabial cyst as a rare case report. Turk Arch Otorhinolaryngol. (2016) 54(2):79–81. 10.5152/tao.2016.1356 29392022 PMC5782938

[19] Swain SK Dubey D. Managing nasolabial cyst: experience at a tertiary care teaching hospital in eastern India. J Clin Sci Res. (2023) 12(4):237–41. 10.4103/jcsr.jcsr_138_22

[20] Zografos I Podaropoulos L Malliou E Tosios KI. Nasolabial cyst: a case report. Oral Surg. (2019) 12(1):51–6. 10.1111/ors.12365

[21] Marcoviceanu MP Metzger MC Deppe H Freudenberg N Kassem A Pautke C Report of rare bilateral nasolabial cysts. J Craniomaxillofac Surg. (2009) 37(2):83–6. 10.1016/j.jcms.2008.11.006 19121949

[22] Sushruth N Prachi N Kush P Singh S Asha K. Nasolabial cyst in an unusual location within the upper lip—a rare case report. Adv Hum Biol. (2022) 12(3):326–8. 10.4103/aihb.aihb_130_21

[23] Ali K Akhtar M Munir M Chatha M. Nasolabial cyst in a child. a case report and review of literature. Eur J Pediatr Dermatol. (2014) 24(1):31–3. Available online at: https://ejpd.com/index.php/journal/article/view/972

[24] Liu E Kridel R. Evaluation of nasoalveolar cysts for the facial plastic surgeon. Arch Facial Plast Surg. (2003) 5(2):185–8. 10.1001/archfaci.5.2.185 12633212

[25] Abdelaziz A Abdelhak B Abdelmoneim R Talaat M. Nasolabial swellings as a rare challenging diagnosis. Minia J Med Res. (2023) 34(4):12–7. 10.21608/mjmr.2023.245843.1531

[26] Bijou W Laababsi R Mennouni MA Oukessou Y Rouadi S Abada R An atypical presentation of a bilateral nasolabial cyst: a case report. J Surg Case Rep. (2021) 2021(3):rjab017. 10.1093/jscr/rjab017 33732421 PMC7947978

[27] Choi JH Cho JH Kang HJ Chae SW Lee SH Hwang SJ Nasolabial cyst: a retrospective analysis of 18 cases. Ear Nose Throat J. (2002) 81(2):94–6. PMID: . 11868481

[28] Zhang J Wu X Ma J. A new transnasal approach of nd:yAG laser treating nasolabial cysts. Lasers Med Sci. (2022) 37:1321–4. 10.1007/s10103-021-03394-y 34379223

